# Pericytes of Stria Vascularis Are Targets of Cisplatin-Induced Ototoxicity: New Insights into the Molecular Mechanisms Involved in Blood-Labyrinth Barrier Breakdown

**DOI:** 10.3390/ijms232415790

**Published:** 2022-12-13

**Authors:** Carmelina Daniela Anfuso, Alessia Cosentino, Aleksandra Agafonova, Agata Zappalà, Giovanni Giurdanella, Angela Trovato Salinaro, Vittorio Calabrese, Gabriella Lupo

**Affiliations:** 1 Section of Medical Biochemistry, Department of Biomedical and Biotechnological Sciences, School of Medicine, University of Catania, 95123 Catania, Italy; 2 Section of Physiology, Department of Biomedical and Biotechnological Sciences, School of Medicine, University of Catania, 95123 Catania, Italy; 3 Faculty of Medicine and Surgery, University of Enna “Kore”, 94100 Enna, Italy

**Keywords:** cochlear pericytes, stria vascularis, cisplatin, phospholipase A_2_, inflammation, VEGF, PDGF-BB

## Abstract

The stria vascularis (SV) contributes to cochlear homeostasis and consists of three layers, one of which contains the blood-labyrinthic barrier (BLB), with a large number of bovine cochlear pericytes (BCPs). Cisplatin is a chemotherapeutic drug that can damage the SV and cause hearing loss. In this study, cell viability, proliferation rate, cytotoxicity and reactive oxygen species production were evaluated. The protein content of phospho-extracellular signal-regulated kinases (ERK) 1/2, total ERK 1/2, phospho-cytosolic phospholipase A_2_ (cPLA_2_), total cPLA_2_ and cyclooxygenase 2 (COX-2) and the release of prostaglandin E2 (PGE2) and vascular endothelial growth factor (VEGF) from BCPs were analyzed. Finally, the protective effect of platelet-derived growth factor (PDGF-BB) on BCPs treated with cisplatin was investigated. Cisplatin reduced viability and proliferation, activated ERK 1/2, cPLA_2_ and COX-2 expression and increased PGE2 and VEGF release; these effects were reversed by Dexamethasone. The presence of PDGF-BB during the treatment with cisplatin significantly increased the proliferation rate. No studies on cell regeneration in ear tissue evaluated the effect of the PDGF/Dex combination. The aim of this study was to investigate the effects of cisplatin on cochlear pericytes and propose new otoprotective agents aimed at preventing the reduction of their vitality and thus maintaining the BLB structure.

## 1. Introduction

The stria vascular (SV), a highly vascularized tissue located in the lateral part of the cochlea, has the function of contributing to cochlear homeostasis [1,2,3]. Because of this, in the SV there are ion transport proteins that maintain, in the endolymph of the cochlear duct, a high concentration of K+ ions and a low concentration of Na+ ions, which contrasts with the opposite concentrations present in the perilymph. This contrasting ionic composition between endolymph and perilymph creates a difference in electrical potential between the cochlear fluids (SV is also referred to as a cochlear battery) called the endo-cochlear potential, which is necessary for the transduction process of hair cells and therefore for auditory function [4].

The SV consists of three layers: a marginal cell layer, exposed to the endolymph, a basal cell layer associated with the fibrocytes of the spiral ligament via junction proteins, with the function of controlling the flow of ions in the SV, and a third layer, intermediate between the first two, which contains the blood-labyrinthic barrier (BLB) [5]. This intermediate layer forms a protective barrier that regulates the passage of molecules from the vascular network of the SV to the cochlear endolymph [6]. Anatomically, the BLB is made up of endothelial cells (EC) connected to each other through tight junctions (TJ), which are mainly responsible for the selective flow of ions and nutrients from the bloodstream to the SV. Other cell types that make up the BLB are pericytes (PCs), closely associated with EC and wrapped together with them by a basement membrane, and the resident perivascular macrophages [3,7]. The three cell types form the vascular cochlear unit [5].

Cochlear PCs are pluripotent cells [8] that regulate blood flow thanks to their contractile properties and contribute to the inflammatory response of the cochlea in numerous pathologies such as sudden sensorineural hearing loss and acoustic trauma [9]. Cochlear PCs are divided into two cell families, the PCs of the SV and PCs of the spiral ligament. These latter express the contractile proteins αSMA and tropomyosin [8]. PCs of the SV express platelet-derived growth factor receptor-β (PDGFR-β), neural/glial antigen 2, (NG2) and are particularly rich in desmin fibers, which confer mechanical strength to the vascular network [5]. They also play an important role in angiogenesis and in the regulation of endothelial TJ protein expression [10]. It has been shown that, after strong sound damage, PCs lose their close association with ECs and detach themselves from SV microcapillaries [11]. Another mechanism by which PCs regulate vessel stability is via pericyte-derived vascular endothelial growth factor (VEGF) [12]. It has been demonstrated that pericytes, induced by VEGF isoform A165 (VEGFA), drive new vessel growth in both adult and neonatal mouse inner ear tissue, thus restoring vascular function in the damaged cochlea [13].

Platelet derived growth factor (PDGF) is an EC-secreted chemokine that binds to the PDGF-β receptor on the plasma membrane of PCs [14]. The binding of PDGF-β to its receptor triggers a signaling pathway that leads to the recruitment of PCs and the formation of the microcapillaries that form the BLB [13]. It has also been shown that PDGF-β is essential for maintaining PC vitality and that inhibition of its release causes the detachment of PCs from microcapillaries [15].

The BLB can be compared to the blood-brain barrier (BBB), as both barriers are formed by a network of microcapillaries formed by ECs sealed together by TJ, which reduce paracellular diffusion and prevent the infiltration of toxic substances or pathogens from the blood to the cochlea or brain [16].

The two barriers differ in their permeability as the BLB is permeable to molecules with a molecular weight up to 500 KDa and to antibiotics such as tobramycin and gentamicin [17], unlike the BBB, which can be crossed by smaller lipophilic molecules and cannot be crossed by tobramycin and gentamicin [18]. Furthermore, astrocytes integrate the neuro-vascular unit of the BBB [19], while perivascular resident macrophage-like melanocytes are present in the cochlear-vascular unit [20].

Although there are strong differences in the permeability between the two barriers, both can undergo pathophysiological changes that could induce exhaustion. The integrity of the barriers is therefore critical to ensure a functionally stable environment [3].

Hearing loss is one of the most common sensory disorders in the human population [21]. It can be caused by numerous conditions such as aging, acoustic trauma from noise, genetic diseases, infections, and exposure to various ototoxic drugs, including cisplatin [22,23]. Unfortunately, the cells of the cochlea have a low regenerative capacity and this makes hearing loss irreversible when the cochlea is damaged. This is a particularly serious condition in children who are undergoing cancer therapy with cisplatin as it can reduce learning ability and delay language acquisition [24]. Therefore, knowing the mechanisms that keep the cochlea functioning can protect hearing ability.

Cisplatin is a chemotherapeutic drug that is frequently used to treat malignant solid tumors [25], capable of damaging the cells that make up the SV, causing serious side effects, in particular permanent and bilateral hearing loss [26]. This condition cannot be prevented by administering otoprotective drugs that could neutralize the anticancer effects of cisplatin [27]. 

Although low reactive oxygen species (ROS) levels during the developmental process have been shown to stimulate the expression of antioxidant defenses [28], there is no doubt that ROS play important roles in a number of diseases, including stroke, traumatic brain injury, and myocardial infarction [29,30]. Cisplatin’s ototoxicity is mediated by the production of oxygen free radicals and by the activation of signaling pathways that trigger the inflammatory process [31,32].

Inflammation is a process that begins with the phosphorylation/activation of cytosolic phospholipase A_2_ (cPLA_2_), catalyzing the arachidonic acid (AA) release from the membrane phospholipids, particularly phosphatidylcholine. AA is converted into prostaglandin (PGs) or leukotrienes (LTs) by the action of cyclooxygenase 2 (COX-2) and 5-lipoxygenase, respectively [33]. During its catalytic cycle, COX-2 generates free radicals and PGs, both with marked pro-inflammatory characteristics [34]. It has been shown that active phospho-extracellular signal-regulated kinases (ERK) 1/2 (p-ERK 1/2) phosphorylates cPLA_2,_ thus activating it, initiating the cascade of molecular events that lead to the production of inflammatory prostaglandin E2 (PGE2) [35,36].

It has been shown that the basal and physiological release of PGs, mediated by COX-1 activity, performs the important function of regulating blood flow in the inner ear [37].

Dexamethasone (Dex) is a steroid drug with a strong anti-inflammatory effect on cochlear tissues, capable of significantly reducing hearing loss when applied locally or systemically [38]. Dex has been shown to preserve hearing in guinea pig models of cochlear implantation [39], and at present, intra-tympanic injections of Dex are very effective in treating sudden deafness caused by circulatory disorders [39]. However, some studies highlight the negative side effects of Dex on inner ear tissues, including increased hearing loss [40].

Since hearing loss is an emerging pathology due the conflicting data on the effect of Dex, which represents the therapy of choice in the treatment of hearing disorders, it is of fundamental importance to investigate the molecular mechanisms involved in hearing loss following damage to the cells of the SV.

Few data are present in the literature on the effects of a therapy based on PDGF. The association of this with Dex, within a collagen matrix, induced the regeneration of the periodontium in monkeys. In particular, it induced new ligament formation, cement deposition and alveolar bone augmentation, demonstrating that the PDGF/glucocorticoid association could represent a new therapeutic agent for the regeneration of periodontitis lesions associated with bone defects [41]. Furthermore, it has been demonstrated that PDGF is capable of inducing a significant proliferation of pulp fibroblasts in vitro, a condition improved by the presence of Dex [42]. Another study demonstrated that Dex increased the proliferation of mouse lung fibroblasts by upregulating the mRNA coding for the PDGF-α receptor, thus producing the A chain of PDGF, which binds to the α-receptor [43].

To date, there are no studies on the effect of PDGF on cells that make up the SV, nor on PDGF-based therapies for pathologies characterized by hearing loss. However, this aspect, aimed at “restoring vitality” to an “offended” cell type, is emerging today. An example is provided by an interesting study that evaluated the possibility of a transplant of “new pericytes” to prevent cisplatin-induced vascular atrophy [44].

The purpose of our study is twofold:

(1) to evaluate the effect of cisplatin on bovine cochlear pericytes (BCPs), which anatomically participate in BLB formation, both in the absence and in the presence of dexamethasone;
(2) to suggest further studies on a new molecule, PDGF-β, for a future therapy based on the restoration/maintenance of the vitality of the pericytes that make up the SV.

## 2. Results

### 2.1. High Cisplatin Concentrations Affected Cell Viability

**Figure 1:**
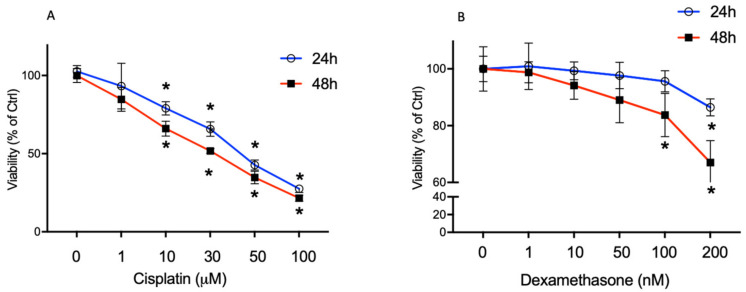
The effect of the treatment for 24 h and 48 h with the increasing concentration of cisplatin (1–100 µM) (**A**) and dexamethasone (1–200 nM) (**B**) on BCPs was evaluated by MTT assay (Cell viability). The lethal concentration (LC) of cisplatin was 50 μM and the LC of Dex was 300 nM. The values are expressed as the mean ± SD of results from three independent experiments, with four parallel samples for each group in each experiment. * *p* < 0.05 vs. cells without treatment at the same incubation time. The non-parametric Mann-Whitney test was used for pairwise comparisons.

Preliminary experiments were performed to assess the subtoxic concentrations for cisplatin and Dex in BCPs. Cell viability was evaluated by MTT assay after treatment of BCPs with 1, 10, 30, 50 and 100 µM cisplatin or with 1, 10, 50, 100 and 200 nM Dex for 24 h and 48 h. In Figure 1A, the treatment of BCPs with 10, 30 and 50 µM cisplatin significantly reduced cell viability by about 21%, 34% and 57%, respectively, after 24 h and by 34%, 47% and 65%, respectively, after 48 h. 

The 100 µM concentration caused a dramatic reduction in the vitality of BCPs both after 24 h and 48 h of incubation.

In Figure 1B, Dex 100 nM reduced cell viability by 26% at 48 h of incubation; Dex 200 nM significantly reduced cell viability by 13% and 33% after 24 h and 48 h of incubation respectively, while at lower concentrations no significant changes were observed.

### 2.2. Dexamethasone Elicits a Protective Effect on Cisplatin-Treated BCPs

Based on our results, the experiments were carried out by treating BCPs with cisplatin 30 µM for 8 h, 24 h, 32 h and 48 h in the absence and in the presence of 10 nM Dex.

**Figure 2:**
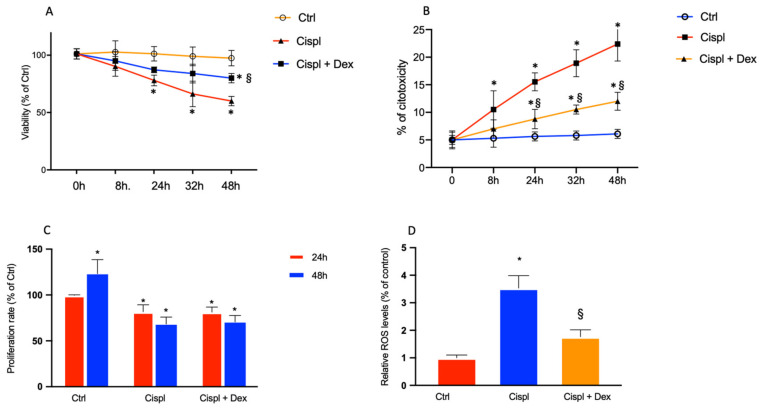
Effects of the treatments for 8 h, 24 h, 32 h and 48 h with 30 µM cisplatin in the absence or in the presence of 10 nM dexamethasone on BCPs were evaluated by MTT assay (cell viability) (**A**) and LDH release (cytotoxicity) (**B**). Effects of the treatments for 24 h and 48 h with 30 µM cisplatin in the absence or in the presence of 10 nM dexamethasone on BCPs were evaluated by crystal violet assays (proliferation rate) (**C**) and an H2DCFDA assay (ROS levels) (**D**); each treatment is represented by a different color: control cells are red, cisplatin-treated cells are blue and cells treated with cisplatin plus dexamethasone are orange. The values are expressed as the mean ± SD of results from three different experiments, with four parallel samples for each group in each experiment. * *p* < 0.05 vs. cells without treatment at the same incubation time; § *p* < 0.05 vs. cells treated with cisplatin. The non-parametric Mann-Whitney test was used for pairwise comparisons.

In Figure 2A, the treatment of BCPs with 30 µM cisplatin significantly reduced cell viability after 24 h, 32 h and 48 h by about 22%, 33% and 40%, respectively, in comparison with control cells. The presence of 10 nM Dex during the treatment of BCPs with 30 µM cisplatin for 24 h, 32 h and 48 h induced a significative increase of cell viability by about 10% 21% and 25%, respectively, in comparison with cells treated with cisplatin in the absence of Dex. In Figure 2B, cytotoxicity, after incubation of BCPs with cisplatin, was evaluated by the determination of LDH release. Cisplatin induced an increase of LDH release by 1.7- and 2.7-fold after 8 h and 24 h, respectively, and by 3.2- and 3.7-fold after 32 h and 48 h, respectively.

The presence of 10 nM Dex during the treatment of BCPs with 30 µM cisplatin for 24 h, 32 h and 48 h induced a significative reduction of LDH release by about 1.8-fold in comparison with cells treated with cisplatin in the absence of Dex.

Cisplatin 30 µM had a pronounced effect on cell proliferation by 18% and 55% after 24 h and 48 h, respectively, in comparison with control cells at the same incubation period. The presence of Dex in the incubation medium containing cisplatin did not have any effect on BCP proliferation, which was maintained at the same values as the cells treated with cisplatin without Dex (Figure 2C). In Figure 2D, ROS levels in BCPs after cisplatin treatment were increased by 3.5-fold in comparison with untreated cells, and Dex reduced their levels by 2-fold.

### 2.3. Dexamethasone Up-Regulates the ERK1/2/cPLA_2_/COX-2 Axis in BCPs Treated with Cisplatin

Because it has been demonstrated that the in vivo treatment of mice with cisplatin is associated with inflammation [21], we assessed the possible involvement of ERK 1/2 and cPLA_2_ in our in vitro model. It is known that the activation of cPLA_2_ by phosphorylation is the necessary event for it to catalyze the release of arachidonic acid from membrane phospholipids and that this process involves ERK 1/2 [35]. 

**Figure 3:**
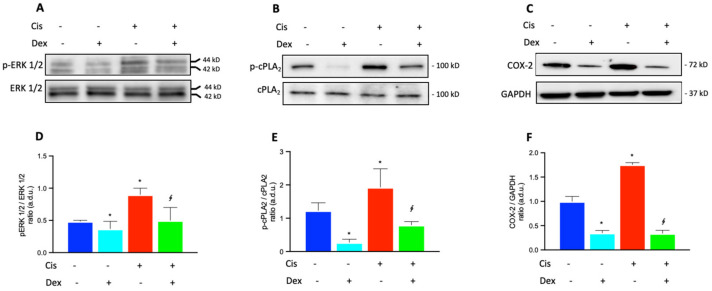
Immunoblot analysis was performed using specific antibodies against p-ERK 1/2 and total ERK 1/2 (**A**), activated (phosphorylated, p-cPLA_2_) and total cPLA_2_ (**B**), and COX-2 (**C**) proteins. The blots were probed with anti-GAPDH (reference) antibody to verify the equal loading of 30 μg protein per lane. Image J software Version 1.52a, NIH, Bethesda, MD, USA). was used to carry out a densitometric analysis of the immunoblots, indicating protein quantification of each band (in arbitrary densitometry units, a.d.u.). Quantitative analysis of western blots for the phosphorylation rate of ERK 1/2 (ratio of p-ERK 1/2/ERK 1/2 in (**D**)) and cPLA_2_ (ratio of p-cPLA_2_/cPLA_2_ in (**E**)) are indicated. Quantitative analysis of western blots for COX-2 protein was normalized to GAPDH (**F**). (**D**–**F**): each treatment is represented by a different color: control cells are blue, dexamethasone-treated cells are light blue, cisplatin-treated cells are red and cells treated with cisplatin plus dexamethasone are green. The data are representative of three independent experiments with four parallel samples for each group in each experiment and are expressed as mean ± SD. * *p* < 0.05 vs. cells without treatment; ^∮^ *p* < 0.05 vs. cells treated with cisplatin. The non-parametric Mann-Whitney test was used for pairwise comparisons.

Immunoblots for p-ERK 1/2 and total ERK 1/2 are reported in Figure 3A. The treatment with 30 µM cisplatin caused a significant increase in the level of phospho-ERK 1/2 by about 1.9-fold in comparison with control cells, as reported in Figure 3D (p-ERK 1/2/ERK 1/2 ratio). The presence of Dex 10 nM reduced the phosphorylation rate of ERK 1/2 by about 44% in comparison with cisplatin-treated cells. No significant effect on p-ERK expression was found in Dex-treated cells in comparison with control cells. 

In Figure 3B, immunoblots for p-cPLA_2_ and total cPLA_2_ are reported. In Figure 3E, the active/phosphorylated form of cPLA_2_ significantly increased by 58% in BCPs treated with cisplatin (p-cPLA_2_/cPLA_2_ ratio) in comparison with control cells. The presence of Dex in the culture medium of cisplatin-treated cells induced a significant decrease of p-cPLA_2_ expression by about 57% and an even more significant reduction of 83% was found in comparison with untreated control cells. The cisplatin-induced increase in cPLA_2_ phosphorylation supports the increase in enzyme activity and therefore a greater release of arachidonic acid is available for cyclooxygenase activity. Based on this assumption, we analyzed COX-2 protein levels after treatment of BCPs with cisplatin in the absence and in the presence of Dex. 

In Figure 3C, immunoblots for COX-2 and GAPDH are reported. In cells with no treatment, levels of COX-2 were elevated (Figure 3C), showing that high amounts of prostaglandins are physiologically produced in BCPs. As reported in Figure 3F, the quantitative analysis of immunoblots for COX-2, expressed by the COX-2/GAPDH ratio, showed that the treatment with cisplatin induced a further increase by 75% compared with untreated control cells, confirming the inflammatory effect of cisplatin. Dex significantly reduced COX-2 levels by 65% and 83% in comparison with untreated and cisplatin-treated cells, respectively.

### 2.4. Dexamethasone Affected COX-2 Fluorescence in BCPs Treated with Cisplatin

**Figure 4:**
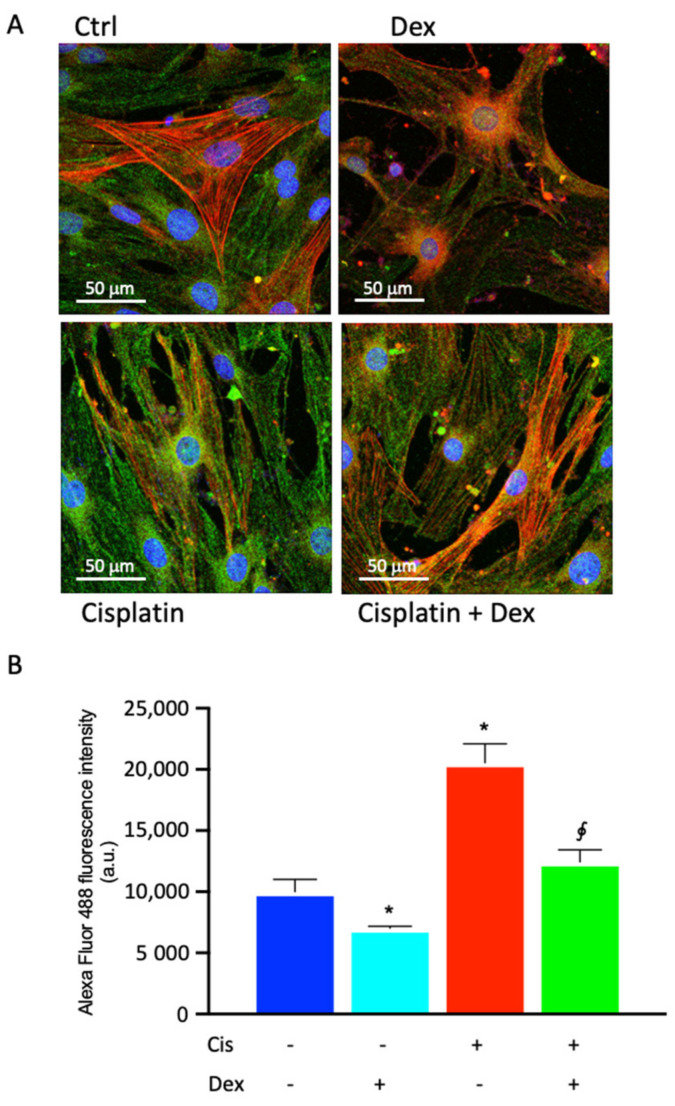
Photomicrographs of COX-2 immunostaining (**A**) of BCP cultures in basal medium, without any treatment (control cells, Ctrl) or in medium containing 30 µM cisplatin in the absence or in the presence of 10 nM dexamethasone (Dex) for 48 h. (**A**) Representative images of immunocytochemical staining for COX-2 (Alexa Fluor 488, green) and alpha smooth muscle actin (α-SMA, Alexa Fluor 546, red) are shown. Blue fluorescence indicates DAPI staining of cell nuclei. Magnification: 40×; Scale bars: 50 μm. (**B**) Quantitative analysis of COX-2 emission intensity (a.u.). In panel (**B**), each treatment is represented by a different color: control cells are blue, dexamethasone-treated cells are light blue, cisplatin-treated cells are red and cells treated with cisplatin plus dexamethasone are green. The data are representative of three independent experiments with three parallel samples for each group in each experiment and are expressed as mean ± SD. * *p* < 0.05 vs. cells without treatment; ^∮^ *p* < 0.05 vs. cells treated with cisplatin. The non-parametric Mann-Whitney test was used for pairwise comparisons.

As COX-2 expression increase in BCPs treated with cisplatin was demonstrated by western blot analysis, and immunostaining was performed to further evaluate COX-2 expression. Cells not treated (control) or treated with cisplatin, in the absence or in the presence of Dex, were marked for α-smooth muscle actin (red) to highlight the cell architecture of the BCPs, and for COX-2 (green), to mark the variations of expression of the enzyme. The merge of the acquisitions, relative to the different treatments, are shown in Figure 4A. The emission intensities of FITC were also evaluated and the pixel values inside the cells, reported in the graph for quantitative analysis of COX-2 (Figure 4B), revealed that the enzyme was highly expressed and uniformly distributed within the untreated cells (control), confirming that it is physiologically very active in BCPs. The treatment with cisplatin increased COX-2 fluorescence by two-fold in comparison with untreated cells and the presence of Dex induced a decrease of COX-2 fluorescence by about 30% and 40% in comparison to untreated and cisplatin-treated cells, respectively. 

### 2.5. Prostaglandin Production and VEGFA Release in BCPs Stimulated and Not Stimulated by Cisplatin in the Absence or in the Presence of Dexamethasone

Since cisplatin treatment significantly induced COX-2, PGE2 production in cisplatin-treated BCPs was quantified. Furthermore, given that VEGF released by PCs has been shown to play a critical role in maintaining vascular function and hearing in the inner ear [13], we also quantified the release of VEGF by BCPs following treatment with cisplatin, in the absence and in the presence of Dex (Table 1). 

Dexamethasone reduced PGE2 production in untreated cells by 48% in comparison with control cells, confirming its suppressing effect on inflammation, already demonstrated by several studies [38,45]. Cisplatin induced an increase of 2.9-fold in comparison with control cells, and Dex significantly reduced the cisplatin-induced release by 2.1-fold.

The incubation of BCPs with Dex resulted in a 22% reduction in VEGF release compared with control cells, whereas a significant increase of 3.1 fold was seen after treatment with cisplatin. Dex reduced VEGF release by almost 41% in comparison with cells treated with cisplatin.

### 2.6. Protective Effect of PDGF-BB on BCPs Treated with Cisplatin

**Figure 5:**
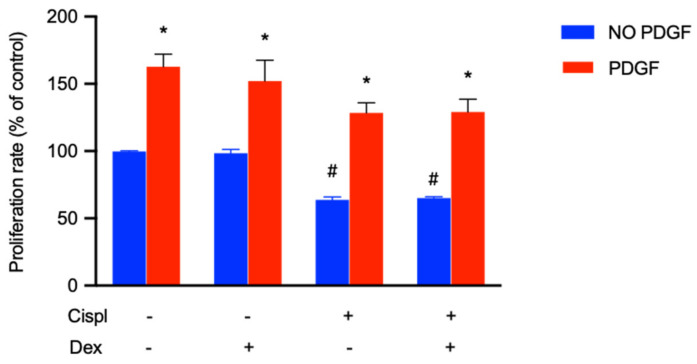
The effect of the treatment for 48 h with PDGF-BB (10 ng/mL) in the presence of cisplatin (30 µM) with or without dexamethasone (10 nM) on BCPs was evaluated by MTT assay. The values are expressed as the mean ± SD of results from three independent experiments, with four parallel samples for each group in each experiment. * *p* < 0.05 vs. cells with the same treatment in the presence of PDGF; # *p* < 0.05 vs. control cells (no treated cells) without PDGF. The non-parametric Mann-Whitney test was used for pairwise comparisons.

Since PDGF is likely to regulate the survival of pericytes [19], and since it has been demonstrated that the PDGF-BB/PDGFR-β signaling pathway had a stimulatory effect on the proliferation of retinal microvascular pericytes [14], experiments were performed by treating BCPs with cisplatin with/without Dex in the presence of 10 ng/mL PDGF for 48 h. In Figure 5, incubation of BCPs with PDGF-BB increased the proliferation rate by about 60%, both in the absence and in the presence of Dex. Moreover, PDGF-BB was able to increase cell proliferation by about two-fold in cisplatin and cisplatin plus dexamethasone-treated cells. These findings make PDGF-BB a pivotal factor in the recovery from cisplatin-induced damage.

## 3. Discussion

A physiologically functioning cochlear microcirculation guarantees a suitable cochlear blood flow to maintain ionic balance and, therefore, the endo-cochlear potential. In fact, an increase in the permeability of SV microcapillaries represents a trigger and aggravating factor in various pathologies characterized by hearing loss, including Meniere’s disease [46]. SV microcapillaries, therefore, represent a district with a key role for inner ear health and the understanding of the mechanisms that regulate the response following damage with cisplatin is essential for choosing the right treatment to deal with hearing pathologies. Cochlear capillaries contain a large number of PCs, with a PC to EC ratio of 1:2, similar to that existing in the retina [8]. Despite their large number, few studies have been conducted on cochlear PCs, and the role they play in maintaining cochlear homeostasis is poorly understood.

The results we obtained indicate that the treatment of BCPs with cisplatin 30 µM for 24 h and 48 h determines a significant reduction in cell viability and proliferation, and a significant cytotoxic effect. Thus BCPs, physically associated with ECs, which control TJs among ECs and stabilize SV microcapillaries, are susceptible to cisplatin damage. This is the first report aimed at investigating the potential damage induced by cisplatin on BCPs.

The presence of Dex induced a slight but significant increase in vital parameters, confirming the protective role played by Dex, already demonstrated by studies conducted on ECs that make up the BBB [47,48]. The BBB differs from the BLB on the basis of the district in which it is located, but has many similarities with the BLB, in the composition of microcapillaries and in the relationships between ECs and PCs.

As demonstrated by numerous studies, there is a close correlation between ROS production and cytotoxicity by cisplatin, which can promote an increase of the levels of free radicals, particularly ROS, to induce damage to DNA, proteins and lipids leading to cancer cell apoptosis [31,49,50,51]. Our results lead to two considerations:

(i) cisplatin induced ROS formation also in BCPs, and therefore the production of ROS, which contributes to making cisplatin very efficient against cancer cells, also has negative effects on non-cancer cells; this is undoubtedly one of the side effects of this chemotherapy;
(ii) Dex, used to counteract cisplatin-induced hearing loss by reducing free radical levels, as we have shown here, could represent one mechanism that reduces the anticancer effects of cisplatin. Indeed, Dex has been shown to reduce the anticancer effects of a drug [27], and can create resistance to chemotherapy through different mechanisms, for example by increasing the adhesion of human ovarian cancer cell lines to the extracellular matrix [52] or by up-regulating Krüppel-like factor 5 in triple-negative breast cancer [53]. The reduction in free radical production induced by Dex could be another mechanism that reduces the antitumor activity of cisplatin.

The correlation between cisplatin and inflammation has already been demonstrated [21], and it has been confirmed in this study. We demonstrated that in BCPs treated with cisplatin, the activation of p-ERK 1/2 leads to the subsequent phosphorylation and therefore activation of cPLA_2_, which releases arachidonic acid. The presence of arachidonic acid activates COX-2 (as demonstrated by the increase in its expression), which transforms arachidonic acid into PGE2 mediators of inflammation. As expected, Dex reduced the levels of p-ERK 1/2, p-cPLA_2_ and COX-2, confirming its anti-inflammatory effect.

COX-2 expression in BCPs without any treatment was very high, and this led us to speculate that COX-2 could be responsible for the basal production of vasodilatory PGE2, which performs the function of maintaining cochlear homeostasis, modulating blood flow in the inner ear [54,55]. When an inflammatory process occurs, COX-2 could produce even larger quantities of PGE2, as already demonstrated by in vivo studies conducted on the organ of Corti and on the spiral ganglion cells of guinea pig cochlea [56].

The high activity of COX-2 is confirmed by the quantification of PGE2, reported in Table 1, whose concentration is already very high in untreated cells and significantly increased after treatment with cisplatin.

To the best of our knowledge, this is the first report that demonstrates that COX-2 is highly expressed in BCPs, which could therefore play a role in the exhaustion of the SV after exposure with cisplatin due to the following events: (i) the cytotoxic action of cisplatin reduces BCP vitality and would lead to their detachment from the ECs of the microcapillaries; and (ii) the loss of BCPs would cause a sharp drop in the concentration of PGE2, which would no longer perform their function of controlling microcapillary permeability.

Therefore, through the activity of COX-2, BCPs could act as “sentinels” responsible for controlling blood flow in the SV. This feature would give BCPs a role that goes beyond that of a simple structural role related to microcapillary-wall integrity [8]. Modulation of COX-2 expression following damage to the inner ear has already been shown. In vivo experiments on guinea pigs exposed to tone bursts demonstrated that COX-2 expression responded differently to noise exposure in the various cochlea districts [57].

It would be interesting (and this will be our next step) to confirm these data with in vitro experiments on an EC/BCP co-culture model which could highlight the different relationships between the two cell types which make up SV microcapillaries, after treatment with cisplatin.

Furthermore, it is of particular importance that the reduction of COX-2 expression after BCP treatment with Dex, in the absence or in the presence of cisplatin, is evident in all the results presented, from the COX-2 protein expression to the release of PGE2 and even in fluorescent images. The effect of Dex on BCPs would lead to a reduction in the production of PGE2, causing damage in the modulation of the vasodilation of the SV microcapillaries.

It has been demonstrated that there is a correlation between the number of PCs and vessel stability; brain and retina microcapillaries, which have the highest density of PCs, have the lowest turnover rate [58]. One of the mechanisms by which PCs regulate vessel stability in the various barriers of our organism is the production of VEGF, which may act in a juxta-crine/paracrine manner as a stabilizing factor of microcapillaries [12].

The significant increase in VEGF production after cisplatin treatment, demonstrated in our in vitro model, could cause an imbalance of VEGF concentration and, given its strong permeabilizing effect, could induce the reduction of endo-cochlear potential and of the expression of tight junction proteins in the SV. Thus, structural and molecular changes in cochlear microcapillaries would be involved in BLB breakdown following exposure to cisplatin. 

Our results demonstrated the effect of Dex on the reduction of VEGF release by both untreated and cisplatin-treated BCPs for the first time, confirming the correlation between inflammation and VEGF release [59]. BCPs, by regulating BLB permeability, could therefore represent a target for the treatment of pathologies associated with hearing loss. 

Dex has been shown to decrease VEGF levels in the astrocytes and pericytes of the BBB, suggesting this as one of the mechanisms by which glucocorticoid treatment can stabilize the BBB [60].

Dex, administered systemically or locally, is widely used in the treatment of pathologies associated with hearing loss, and numerous studies have been conducted to evaluate different routes of intra-cochlear administration and transport [61]. Administration with intratympanic injection can obtain an optimal concentration of the drug in the cochlea, avoiding the physiological reduction of its bioavailability in the inner ear [62]. For an atraumatic treatment, several studies have evaluated its transport within different kinds of nanoparticles, comparing them with each other [45]. Another factor to be taken into consideration for the design of innovative delivery systems for glucocorticoids is their low solubility in an aqueous environment and the fact that their receptors are intracellular [63].

Although Dex is widely used for the treatment of diseases characterized by hearing loss, including sudden sensorineural hearing loss (SSNHL) [39] and Meniere’s disease [64], there are in vivo studies showing that Dex had no effect [65] or that there was remarkable heterogeneity in treatment response across patients with SSNHL [66], and some even reported a toxic effect [38,66]. Moreover, to date, a significant knowledge gap exists relating to the localization of the action of corticosteroids in the inner ear and the cell types involved in their response [66]. 

The therapeutic use of molecules that increase BCP survival could avoid or reduce the unpleasant side effects caused by the use of corticosteroids. To this end, we conducted in vitro experiments, treating BCPs with cisplatin in the presence of PDGF-BB, a molecule that promotes their proliferation and migration [14]. The results shown in Figure 5 highlight a recovery of the proliferation of the cisplatin-exposed BCPs. This result opens new frontiers in the study of the treatment of pathologies of the inner ear characterized by the exhaustion of the BLB. 

BCPs play a fundamental role in the cochlea, both from a physiological point of view (regulation of cochlear blood flow, maintenance of the structure of SV microcapillaries by controlling TJ expression between ECs and by producing VEGF) and pathological (PC loss has been demonstrated in numerous cochlear diseases) [9]. Although the BLB is one of the most important targets for delivering therapeutics to the inner ear, there are still few studies that consider it a clinical treatment target. 

A recent study has shown that transplantation of young PCs into mouse cochlea rehabilitates the vascular regression induced by loud sound trauma and improves hearing [44]. 

The results of our study demonstrated the importance of knowing the signaling pathways that are activated in BCPs after treatment with cisplatin. Treatment with molecules that support their vitality would allow the physiological replenishment of vasodilating prostaglandins and the maintenance of the structure of the microcapillaries forming the BLB.

Studies on otoprotective agents, particularly aimed at preventing PC loss, could be a promising and new therapeutic strategy in consideration of the pharmacological limitations of therapies in use today. Finding innovative treatments based on insights into the molecular mechanisms is the gamble of the future for the treatment of diseases with hearing loss.

## 4. Materials and Methods

### 4.1. Reagents

VEGF-A was acquired from Peprotech (Rocky Hill, NJ, USA); PGE2 came from Cayman Chemical (Ann Arbor, MI, USA); mouse monoclonal antibodies against cPLA_2_ were purchased from Santa Cruz (Santa Cruz, CA, USA); mouse monoclonal antibody against GAPDH was purchased from Abcam (Cambridge, UK); rabbit polyclonal antibodies against phospho-cPLA_2_ and COX-2 were purchased from Cell Signaling Technology (Danvers, MA, USA); secondary goat anti-mouse TEXAS RED conjugated antibody was purchased from Calbiochem; and secondary goat anti-rabbit IRDye 680 conjugated antibody was purchased from LI-COR (Lincoln, Dearborn, MI, USA). Cisplatin and dexamethasone were purchased from Sigma-Aldrich (Munich, Germany). Media, antibiotics and other reagents for cell cultures came from Innoprot (Elexalde Derio, Spain).

### 4.2. Cell Culture and Experimental Protocol

Primary bovine cochlear PCs (BCPs) were generously provided by prof. Giuseppe Moltabano of the University of Messina. BCPs were isolated from the stria vascularis of the explanted bovine cochleae as previously described [67], and were fed with pericyte medium supplemented with 5% FBS, bovine serum albumin (BSA) 10 μg/mL, apotransferrin 10 μg/mL, insulin 5 mg/mL, epidermal growth factor 2 (EGF 2) 2 ng/mL, fibroblast growth factor 2 (FGF 2) 2 ng/mL, insulin like growth factor 1 (IGF 1) 2 ng/mL, hydrocortisone 1 mg/mL, 100 U/mL penicillin, and 100 mg/mL streptomycin. 

In a first set of experiments, cells at confluence were incubated for 24 h and 48 h with increasing concentrations of cisplatin (1, 10, 50, 100 and 200 µM) or with dexamethasone (1, 10, 50, 100 and 200 nM). After measurement of cell viability, the concentration of 10 and 30 µM cisplatin alone or in the presence of 10 nM dexamethasone were chosen for the experiments.

In a second set of experiments, cells at confluence were incubated with PDGF-BB (10 ng/mL) for 48 h and, during this period, the treatments with cisplatin 30 µM in the absence or in the presence of dexamethasone 10 nM were carried out. Cell viability was measured at the end of the incubation period.

### 4.3. Cell Viability

To determine cellular viability, the 3-[4,5-dimethylthiazol-2-yl]-2,5-diphenyl tetrasodium bromide (MTT) assay was used (Chemicon, Temecula, CA, USA). Cells were seeded in 96-well plates at 1500 cells/well to obtain optimal cell density throughout the experiment. Cells were incubated at 37 °C for 4 h with MTT (0.5 mg/mL). At the end of incubation, 100 mL isopropanol/0.04N HCl (1:10) was added and the plates were incubated for 1 h at room temperature. The absorbance of each well was measured at 570 nm in a plate reader (VariosKan, Thermo Fisher Scientific, Waltham, MA, USA). 

LDH release was assessed by using a commercial assay (Roche Diagnostics) and expressed in cytotoxicity (%) [(sample absorbance/lysed cell absorbance − control absorbance) × 100].

### 4.4. Cell Proliferation Assay

To evaluate the proliferation rate of BCPs, crystal violet staining was used. After incubation with cisplatin 30 mM in the absence or in the presence of dexamethasone 10 nM for 24 h and 48 h, cells were stained with 0.5% crystal violet solution in 20% methanol for 10 min. Cells were then washed with distilled water and left to dry. Crystal violet was solubilized and absorbance values were measured at 570 nm with a microplate reader (Synergy 2-BioTek). Each assay was carried out in triplicate from three independent experiments.

### 4.5. Western Blotting 

After treatments, BCPs were lysed as previously described [68]. The protein content of the cell lysate was quantified by the Bradford assay, 40 µg proteins were loaded into polyacrylamide gels, run in SDS-PAGE and blotted as described elsewhere [69]. Membranes were incubated with primary antibodies against phospho-cPLA_2_ (1:500 dilution), phospho-ERK 1/2 (1:500 dilution), and cyclooxygenase-2 (COX-2), (1:500 dilution). GAPDH (1:1000) was used as the loading control. The membranes were then incubated with secondary fluorescent antibodies (1:20,000 dilution) for 1 h at room temperature. The immunoblot was detected through an Odyssey Imaging System (LI-COR Biosciences, Lincoln, NE, USA). Densitometry analyses of the blots were performed using Image J software (National Institutes of Health, Bethesda, MD, USA). Membranes used to detect total cPLA_2_ and total ERK 1/2 were stripped and re-probed with phospho-cPLA_2_ (1:500 dilution) and phospho-ERK1/2 (1:800 dilution).

### 4.6. Confocal Microscopy

Before cell seeding, glass chamber slides were placed in a 24-well plate, coated with a 10 mg/mL poly-l-lysine solution (PLL, Innoprot) for 1 h at 37 °C and washed with sterile water. BCPs were seeded onto PLL-coated glass chamber slides at a cell density of 5 × 10^4^ cells/well and incubated at 37 °C in a humidified atmosphere of 5% CO_2_, until confluence. Subsequently, cells were treated with 10 µM cisplatin with or without 10 nM dexamethasone for 48 h.

At the end of the incubation period, cells were fixed with ice-cold acetone for 15 min followed by ice-cold methanol for 20 min and then washed in PBS. Fixed cells were incubated overnight at 4 °C with rabbit COX-2 (1:400 dilution) and a-SMA mouse antibodies (Santa Cruz) (1:100) in PBS/triton 0.1%. After washing with PBS, cells were incubated for 1 h with green goat polyclonal anti-rabbit Alexa 488-conjugated secondary antibodies (1:1000 dilution, Molecular Probes, Cat. No. A-11008) and with red goat polyclonal anti-mouse Alexa Fluor 546-conjugated secondary antibodies (1:1000 dilution, Innovative Research, Cat. No. A21045), at room temperature in the dark. Slides were then mounted using mounting medium (Life Technologies, Carlsbad, CA, USA) and observed using a Leica TCS SP8 microscope. Fluorescence intensity was quantified using ImageJ analysis software (Version 1.52a, NIH, Bethesda, MD, USA).

### 4.7. ROS Measurements

Reactive oxygen species (ROS) were measured by means of the DCFDA—Cellular Reactive Oxygen Species Detection Assay Kit (ab113851, Abcam Cambridge, UK), according to the manufacturer’s protocol [70]. After the treatments, BCPs were incubated with 25 μM 2′,7′-dichlorodihydrofluorescein diacetate (DCFDA) in a buffer solution at 37 °C for 30 min. DCFDA was then replaced with 100 μL of medium and the ROS concentration was measured by VarioskanTM (λex = 495 nm, λem = 529 nm).

### 4.8. Prostaglandin E2 Production and VEGF Release

To determine PGE2 and VEGF release, BCPs were incubated with 30 mM cisplatin in the absence or in the presence of 10 nM dexamethasone for 48 h. Supernatants were collected, and aliquots were used for PGE2 determination using a kit from Cayman Chemicals Co., Ann Arbor, MI, USA. For PGE2, the detection range was 7.8 to 1000 pg/mL [71]. Conditioned medium was also analyzed for VEGF by ELISA, using a kit from R&D Systems Inc., Minneapolis, MN, USA, as specified by the manufacturer’s instructions. For VEGF, the detection range was 20 to 2500 pg/mL. Each sample from three different experiments was analyzed in triplicate.

### 4.9. Statistical Analysis

A total of three experiments were carried out and each experiment included four parallel samples for each group (*n* = 4). The data are reported as the mean ± the standard deviation (SD). The different groups/conditions were compared by the non-parametric Mann-Whitney U-test; a *p* value < 0.05 was considered to denote a statistically significant difference between the experimental and control groups. The statistical analysis and graph design were carried out by means of GraphPad Prism 7.00 software (GraphPad Inc., San Diego, CA, USA).

## 5. Conclusions

In conclusion, the study carried out on BCPs leads to the following considerations:

(1) BCPs are a target of cisplatin damage: the reduction of their viability could cause a decrease in PGE2 production, with severe implications for microcapillary permeability;
(2) one of the mechanisms by which cisplatin performs anticancer activity is the production of ROS, which leads to the death of cancer cells. Dex, in the presence of cisplatin, reduced ROS production and this mechanism could interfere with the antitumor activity of cisplatin;
(3) cisplatin triggered an inflammatory process in BCPs by activating p-ERK 1/2, p-cPLA_2_ and COX-2 and inducing an increase in the release of PGE2. Dex reduced PGE2 production and therefore reduced the modulation of SV permeability;
(4) the treatment of BCPs with PDGF-BB induced a recovery of their proliferation in the presence of cisplatin. The therapeutic use of PDGF-BB could allow for the replenishment of PGE2 and the maintenance of the BLB structure.

## Figures and Tables

**Table 1:** Prostaglandin production and VEGFA release in BCPs stimulated and not stimulated by cisplatin 30 μM in the absence or in the presence of 10 nM dexamethasone for 48 h.

Treatment	PGE2 Release (pg/mL) ± SD	VEGF Release (pg/mL) ± SD
None	78 ± 8.2	41.6 ± 3.8
Dexamethasone 10 nM	40.6 ± 5.3 *	32.3 ± 3.1 *
Cisplatin 30 μM	232.4 ± 21.6 *	131.6 ± 11.4 *
Cisplatin 30 μM + Dexamethasone 10 nM	108.5 9.1 ^§^	78.1 ±6.9 ^§^

Cell culture supernatants from BCPs in the absence (none treatment) and the presence of 30 mM cisplatin, with or without 10 nM dexamethasone, were assayed for PGE2 production and VEGFA release. Values are from three independent experiments (*n* = 3). The non-parametric Mann-Whitney test was used for pairwise comparisons. * *p* < 0.05 vs. cells without treatment; ^§^  *p* < 0.05 vs. cells treated with cisplatin.

## Data Availability

The data presented in this study are available on request from the corresponding author. The data are not publicly available due to reasons of privacy.
